# Insights into the Role of Gremlin-1, a Bone Morphogenic Protein Antagonist, in Cancer Initiation and Progression

**DOI:** 10.3390/biomedicines10020301

**Published:** 2022-01-28

**Authors:** Noha Mousaad Elemam, Abdullah Imadeddin Malek, Esraa Elaraby Mahmoud, Waseem El-Huneidi, Iman M. Talaat

**Affiliations:** 1 College of Medicine, University of Sharjah, Sharjah, United Arab Emirates; noha.elemam211@gmail.com (N.M.E.); abdullahmalek96@gmail.com (A.I.M.); esrahmed@live.com (E.E.M.); 2 Sharjah Institute for Medical Research, University of Sharjah, Sharjah, United Arab Emirates; 3 Faculty of Medicine, Alexandria University, Alexandria 21526, Egypt

**Keywords:** Gremlin-1, BMP antagonist, breast cancer, colon cancer, metastasis, adipokine

## Abstract

The bone morphogenic protein (BMP) antagonist Gremlin-1 is a biologically significant regulator known for its crucial role in tissue differentiation and embryonic development. Nevertheless, it has been reported that Gremlin-1 can exhibit its function through BMP dependent and independent pathways. Gremlin-1 has also been reported to be involved in organ fibrosis, which has been correlated to the development of other diseases, such as renal inflammation and diabetic nephropathy. Based on growing evidence, Gremlin-1 has recently been implicated in the initiation and progression of different types of cancers. Further, it contributes to the stemness state of cancer cells. Herein, we explore the recent findings on the role of Gremlin-1 in various cancer types, including breast, cervical, colorectal, and gastric cancers, as well as glioblastomas. Additionally, we highlighted the impact of Gremlin-1 on cellular processes and signaling pathways involved in carcinogenesis. Therefore, it was suggested that Gremlin-1 might be a promising prognostic biomarker and therapeutic target in cancers.

## 1. Introduction

The cellular pathways responsible for normal cellular growth, differentiation, and survival are aberrant in cancerous cells. The uncontrolled growth of cancer cells is a result of accumulated mutations that alter the cellular regulatory mechanisms leading to the formation of tumors. Tumors are not only composed of neoplastic cells, but several other soluble and cellular components that exist in the milieu, including inflammatory mediators’ tumor-associated cytokines, stromal cells (fibroblasts, endothelial cells and immune cells), and most importantly cancer stem cells (CSCs) [1,2]. CSCs are distinguished by their ability to self-renew and differentiate [3], in addition to resistance to therapy, immune system evasion, ability to invade surrounding stromal tissue and blood vessels, as well as metastasis [4,5]. Several factors seem to affect cancer initiation, progression, and growth. However, the exact mechanism is still not yet understood. Among these factors is the loss of cell–cell adhesion molecules known as cadherins, which are implicated in cancer progression [6,7]. 

Notably, cancer cells orchestrate tumor growth and invasion through interacting with surrounding stromal cells, such as fibroblasts, endothelial cells, and immune cells [8]. Such interactions in the tumor microenvironment (TME) facilitate other processes, such as angiogenesis and metastasis [9]. Angiogenesis is a crucial process that paves the way for tumor growth, invasion, and metastasis that leads to the spread of malignant cells to distant organs [10]. Such complex processes are driven by several genetic and epigenetic factors [11]. Moreover, soluble cytokines released in the TME, such as the transforming growth factor-beta (TGF-β) superfamily (including TGF-β cytokines, activins, and bone morphogenic proteins (BMPs)), regulate tumor growth, metastasis, and function [12]. Gremlin-1, one of the BMP antagonists, is known to play a vital role especially during lung, kidney, and bone development, as well as being a niche factor contributing to cell proliferation by blocking BMP signaling [13,14]. In this review, we aim to shed light on the recent updates and potential role of Gremlin-1 in different types of cancer.

## 2. Gremlin-1

Cytokines are a wide group of proteins that exhibit a crucial role in many physiological and cellular functions. They are classified into different superfamilies, including the TGF-β superfamily [15]. TGF-β has over thirty evolutionary conserved members, including three TGF-β cytokines, four activins, four neurotrophic factors, and 21 BMPs and BMP antagonists [16]. All of these proteins are highly regulated, secreted in a soluble form, and contain a characteristic cysteine knot module. Furthermore, several members of this family bind to structural polysaccharides (heparin and heparin sulfate) which enables them to attach to the extracellular matrix (ECM) and act as a paracrine cytokine [16,17]. 

Furthermore, it has been proposed that the multiple functions exhibited by different members of the TGF-β superfamily could be attributed to differences in the total number of cysteine knot motifs and their capabilities to bind to the ECM polysaccharides [16]. In this context, the Cerberus or DAN (CAN) families, which have eight cystine knot motifs, act as natural antagonists of the BMP family. Although members of this family include Gremlin-1, Gremlin-2/PRDC, Sclerostin, USAG-1, Cerberus, Coco, and Dan, only Gremlin-1, Gremlin-2/PRDC, and Sclerostin are known to bind to heparin and heparan sulfate. This interaction is important not only to prevent protein diffusion, but also to act as a negative regulator of the BMP signaling cascade [17,18].

Similar to other members of the TGF-β family, Gremlin-1 is a protein consisting of 184 amino acids which has a cysteine-knot motif and cysteine-rich region [19]. It is encoded by the *GREM1* gene which was initially discovered in rats under the name of drm [20]. The sequence of *GREM-1* has been substantially conserved throughout evolution and the human *GREM-1* gene was reported to be localized in chromosome 15q13–q15. When it comes to its protein structure, it has been revealed that the first 24 amino acids serve as a signaling peptide while the cysteine-rich region and the cystine knot motifs are located between amino acids 94 and 184. Furthermore, analysis of amino acid sequences revealed multiple potential glycosylation and phosphorylation sites, especially near the N-terminus and C-terminus, that are proposed as important sites for the nuclear localization [21,22].

**Figure 1:**
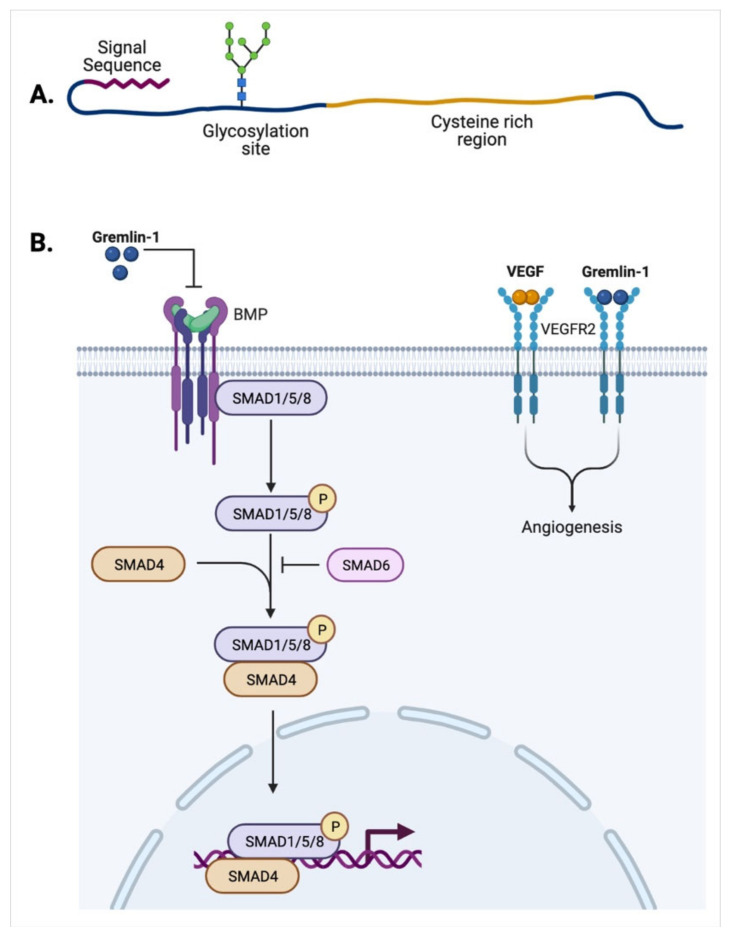
Structure and Function of Gremlin-1. (**A**) Schematic diagram representing the structure of Gremlin-1 with its signal sequence, glycosylation site and cysteine-rich region. (**B**) Gremlin-1 can bind and inhibit the BMP signaling pathways including the SMAD family as well as direct binding to vascular endothelial growth factor receptor (VEGFR) and activating its downstream signaling pathway.

In 1998, Hsu and his colleagues described, for the first time, Gremlin-1 as an antagonist of the BMPs in a *Xenopus* model [23]. Within the BMP signaling pathway, the ligands and their antagonists are highly conserved throughout evolution and play key roles in important processes such as limb generation as well as kidney and bone development [24]. Activation of the canonical signaling pathway requires the binding of BMP ligands to BMP receptor 2 (BMPR2), a transmembrane serine/threonine kinase receptor which consequently binds and phosphorylates BMPR1. This signaling pathway is activated by BMP ligands and leads to the activation of multiple downstream proteins, namely Smad 1, 5, and 8. These proteins interact and form complexes with Smad 4 [25]. Upon the translocation of these complexes into the nucleus, they will act as transcriptional factors that regulate the expression of genes involved in some target pathways such as Smad 6 (Figure 1). On the other hand, when BMP antagonist proteins such as Gremlin-1 bind to BMP receptors, the inactivation of the canonical pathway will occur, thus inhibiting all of its subsequent effects. This is an important regulatory mechanism of the BMP pathway [24]. As a result, when the expression or function of these proteins is defective, processes such as adult tissue homeostasis and embryonic development are disrupted. For instance, it has been reported that some aberrant limb patterning and digitization in mice embryos have been linked to *GREM-1* gene mutation [26]. Additionally, mice with homozygous Gremlin-1 knockout were not able to survive and eventually died after birth [27].

Aside from the role of Gremlin-1 in development, the dysregulation of Gremlin-1 has been observed in different pathological conditions, such as diabetic nephropathy [28,29], pancreatitis [30], osteoarthritis [31], kidney failure [22], as well as liver and lung fibrosis [32]. Additionally, a growing body of evidence suggests that certain cancer types have dysregulated BMP signaling due to a disequilibrium of BMPs and their antagonists. As a result, the interaction with secreted antagonists in BMP pathways such as Gremlin-1 is an essential mechanism of signal regulation. However, the function of Gremlin-1 in cancer pathogenesis is not limited to BMP signaling. Of particular interest is the interaction with its only known receptor vascular endothelial growth factor receptor-2 (VEGFR2) among other cellular interactions in the TME. 

## 3. Gremlin-1 and BMP in Cancer 

The BMP pathway is another molecular “Jekyll and Hyde” of carcinogenesis, as it has dual tumor-promoting and tumor-suppressive activities, depending on the cancer cell type, TME, and the stage of tumor growth [33]. Gremlin-1 was found to be overexpressed in various human tumors, including carcinomas of the lung, ovary, kidney, breast, colon, pancreas, and sarcomas [34]. 

Extracellularly, Gremlin-1 acts as an antagonist to the anti-proliferative effects of BMP-2, BMP-4, and BMP-7 by forming heterodimers and preventing their binding with their respective cell surface receptors. Intracellularly, Gremlin-1 interacts with BMP-4 precursor protein and downregulates its signalling in fetal lung embryogenesis. The signaling of BMPs can induce a myriad of biological responses that are implicated in carcinogenesis and metastasis of cancerous cells [35]. The overexpression of Gremlin-1 was found to increase the level of p21 (Cip1) protein and phosphorylate p42/44 MAPK kinases [36]. The excess of Gremlin-1 is known to inhibit BMP [37], allowing epithelial cells to retain stem cell-like features, develop ectopic crypts, and eventually become cancerous in animal models [38].

It has been reported that Gremlin-1 is highly expressed in the stromal fibroblasts of a multitude of cancer types, suggesting cancer-associated fibroblasts (CAFs) to be a potential source of Gremlin-1 [39,40,41]. This leads to an autocrine activation of fibroblasts, hence enhancing the invasion and stemness of cancer cells as reported in breast cancer [35]. One of the hallmarks of epithelial-mesenchymal transition (EMT) is the loss of E-cadherin expression by cancer cells [42,43]. E-cadherin is regulated by a number of transcription factors, namely Snail and Slug, that facilitate the acquisition of a mesenchymal phenotype and induce EMT [44]. This is observed in patients suffering from breast cancer or oral squamous cell carcinoma where there was an inverse correlation between the patients’ survival and the expression of Snail and E-cadherin [45,46]. BMP-2 is known to attenuate Snail expression. Hence, BMP inhibitors such as Gremlin-1 facilitate the upregulation of the transcription factor Snail and thus promote EMT induction [47,48]. Moreover, studies have shown a decreased expression of E-cadherins in the presence of Gremlin-1 [49]. Despite its preferential interaction with BMP-2, 4, 7 [50], the pro-angiogenic and pro-inflammatory activity of Gremlin-1 seem to be independent of its effects on BMP [51,52].

## 4. Gremlin-1 and VEGFR2 

Vascular Endothelial Growth Factor (VEGF) is a known endothelial-specific growth factor that plays a crucial role in angiogenesis by promoting endothelial cellular proliferation, differentiation and enhancing microvascular permeability and vasodilation [53]. Similar to VEGF, Gremlin-1 is also considered a pro-angiogenic factor. Moreover, both VEGF and Gremlin-1 belong to the cysteine-knot superfamily and bind to VEGFR2, the main transducer of VEGF-mediated angiogenic signals in endothelial cells and the only known surface receptor of Gremlin-1 [54]. This promotes a cascade of intracellular events that stimulate neovascularization and angiogenesis [55]. Alternatively, Gremlin-1 could interact with cancer cell lines that did not express VEGFR2, suggesting another possible mechanism of action of Gremlin-1, elucidating its ability to bind to cancer cells by way of other receptors or mechanisms [56]. 

Covalent and monomeric forms of Gremlin-1 can act as BMP antagonists and are equally effective in inhibiting BMP-mediated intracellular cascade of events. Conversely, they operate in different ways when it comes to binding to VEGFR2. Monomeric Gremlin-1 antagonizes the action of VEGFR2, unlike the covalent Gremlin-1 which activates VEGFR2. Moreover, Gremlin-1 carries anti-angiogenic and anti-tumorigenic functions as its expression in breast and prostate cancers hindered tumor vascularization and growth [55]. Furthermore, in pulmonary hypertension, the levels of monomeric Gremlin-1 secreted by the hypoxic lung cells surged, inhibiting neovascularization, leading to further lung damage, hypoxia, and disease progression [57]. Thus, the Gremlin-1/VEGFR2 axis is considered as a promising therapeutic target that needs to be further studied, due to the fact that the currently approved VEGFR2 inhibitors are associated with side effects, such as hypertension, hypothyroidism, and coagulation disorders [58]. 

## 5. Gremlin-1 and Other Components of Tumor Microenvironment

### 5.1. Matrix Metalloproteinases (MMPs)

**Figure 2:**
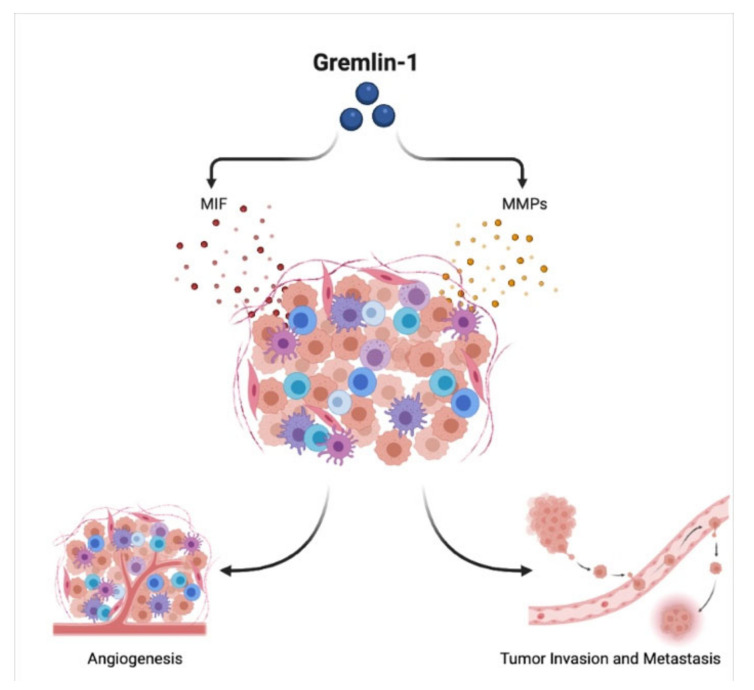
Gremlin-1 and Tumor Microenvironment. Gremlin-1 regulates the expression of tumor microenvironment regulators, migration inhibitory factor (MIF) and metalloproteinases (MMPs), leading to the activation of angiogenesis, tumor invasion and metastasis.

MMPs are a group of proteolytic enzymes that are highly produced in different cancer environments, facilitating tumor proliferation, survival, angiogenesis and finally metastasis by breaking down the ECM and modulating the tumor stroma [59,60]. It is noteworthy that there is a relationship between different subtypes of MMPs and Gremlin-1 in multiple cancers, as shown in Figure 2. For instance, the levels of MMP-13 mRNA in breast cancer cells significantly decreased when Gremlin-1 was knocked down. As a result, the tumor cell proliferation and dissemination were inhibited, which indicated that the Gremlin-1/MMP13 axis hampered the breast tumor growth [61]. 

Another study showed that Gremlin-1 increased the levels of MMP-2 and MMP-14 in mesothelioma cancer cells, resulting in tumor progression and metastasis. These findings shed the light on the possibility of targeting the Gremlin-1/MMP axis as a future therapeutic approach to control and restrict cancer growth [62].

### 5.2. Migration Inhibitory Factor (MIF) and Monocyte Differentiation 

Although this review focuses on the effect of Gremlin-1 in cancerous cells, it is important to note that it also plays a role in the pathogenesis of other conditions. Of significance, Gremlin-1 is proven to be overexpressed in injured endothelial cells, suggesting that this BMP antagonist can play a role in the pathophysiology of atherosclerosis [63]. Eventually, the role of Gremlin-1 in atherosclerosis was established and studies showed that Gremlin-1 inhibits the role of migration inhibitory factor (MIF), a cytokine that is released by different types of body cells and offers a wide range of biological functions. Further, MIF is known to regulate the recruitment of monocytes towards the atherosclerotic lesions, the differentiation of monocytes into macrophages at the plaque site and the secretion of tumor necrosis factor (TNF)-α by the macrophages. Consequently, we can deduce that as Gremlin-1 hampers the action of MIF, and the atherosclerotic process will eventually be suppressed [64].

High levels of MIF were prominently involved in the recruitment and differentiation of monocytes and regulation of inflammation. Additionally, MIF could promote cancer growth, invasion, and angiogenesis in several types of tumors, highlighting MIF as a prognostic biomarker [64,65]. As illustrated in Figure 2, Gremlin-1 was found to be positively correlated with MIF levels in pancreatic ductal adenocarcinoma, and inversely correlated with pro-tumorigenic M2 macrophage phenotype in the cancer microenvironment. Hence, Gremlin-1 indirectly leads to a decreased level of M2 macrophages, thus having an inhibitory effect on the cancer growth [66]. 

## 6. Gremlin-1 and Cancer Types

### 6.1. Breast Cancer

Several studies reported the role of Gremlin-1 in breast cancer progression by assessing cell proliferation, migration, and invasion. For example, the knockdown of *GREM-1* led to a decrease in the proliferation potential of breast cancer cells. Moreover, these cells showed an inhibition in the xenograft mammary tumor growth, as well as its ability to metastasize to lung tissue compared to control cells [61,67,68]. On the contrary, overexpression of *GREM-1* induced growth, migration, and invasion of breast cancer cells [61]. Alternatively, Kim N.H. et al. reported another possible mechanism of action of Gremlin-1 in breast cancer pathogenesis, where *GREM-1* overexpression caused an increase in glucose uptake and lactate production [69]. Further exploration in this metabolic pathway revealed Gremlin-1 to induce the expression of hexokinase-2 (HK2) and activate STAT3, thus catalyzing the phosphorylation of glucose and activating the ROS-Akt signaling pathway, respectively. The Gremlin-1 induced ROS-Akt-STAT3 activation was found to regulate HK2 expression and eventually boost glycolysis in breast cancer cells. Another possible suggestion for Gremlin-1 regulation of cancer metabolism was through the increase in receptor tyrosine kinase activity. Such metabolic dysregulation could lead to breast cancer progression [69].

Gremlin-1 expression was found to be elevated in breast cancer cell lines and tissues obtained from patients. Such overexpression was associated with poor prognosis in breast cancer patients, especially those with negative estrogen receptor (ER) in comparison to ER-positive tumors [68]. A possible link between estrogen and Gremlin-1 was found to be through the direct interaction of estrogen-related receptor α (ERRα) and *GREM-1* promoter, leading to the induction of *GREM-1* expression. This resulted in a positive feedback mechanism where Gremlin-1 stimulated the promoter activity of the gene encoding ERRα, which is known to be essential for the growth of ER-negative breast cancer cells [70]. On another note, Gremlin-1 was found to promote the progression of breast cancer by inducing EGFR signaling pathway, an upstream regulator of ERRα [68]. Therefore, Gremlin-1 can activate the EGFR–ERRα axis and eventually acts as an enhancer for the expression of genes involved in cancer cell growth and proliferation [68]. Interestingly, a recent study by McNamee N. et al. reported that sera of breast cancer patients carrying Gremlin-1 were higher in breast cancer patients compared to healthy controls [71]. This highlights the potential use of these extracellular vesicles as a minimally invasive biomarker tool [71].

*GREM-1* was found to be significantly upregulated in cells and primary tumors of metastatic 66cl4 cell line in comparison to the non-metastatic 67NR of the 4T1 mouse mammary tumor model. In addition, *GREM-1* expression was correlated with the upregulation of stem cell markers in 66cl4 cells compared to 67NR cells [67]. Using an in vivo mice model and human patients, the expression of Gremlin-1 was associated with extracellular matrix organization and formation, as well as collagen biosynthesis and modification [67]. Another study revealed that the high expression of Gremlin-1 in breast cancer stroma is correlated with a poor prognosis regardless of the molecular subtype. Breast CAFs showed high expression of Gremlin-1 both in vitro and in vivo [72]. Looking closely to the TME of breast cancer, it was suggested that the TGF-β and inflammatory cytokines secreted by breast cancer cells stimulated Gremlin-1 expression in CAFs. This would lead to the elimination of BMP/SMAD signaling in breast cancer cells and stimulation of mesenchymal phenotype, stemness, and invasion while inducing the fibrogenic activation of CAFs [72]. Hence, Gremlin-1 was denoted as a key factor in the interplay between breast cancer cells and CAFs, regulating cancer cell invasion [72]. Docosahexaenoic acid, an omega-3 fatty acid abundant in fish oils, was found to reduce the expression of Gremlin-1, leading to an inhibition of p-ERK, mesenchymal cell-associated genes, and cell migration. This shows the possible role of Gremlin-1 in the EMT process in human breast cancer cells [61,73]. Numerous studies agree that Gremlin-1 might be a potential and promising therapeutic target for breast cancer [61,68,72].

### 6.2. Cervical Cancer

On another note, high *GREM-1* expression in cervical cancer tissues was reported to be a prognostic factor of overall survival and correlated with bulky tumor size [74]. Additionally, cervical cancer cells exposed to Gremlin-1 showed an alteration in differentiation cell markers and cancer stem cell-like properties. Thus, Gremlin-1 may have a role in the clinical recurrence of cervical cancer [74].

### 6.3. Basal Cell Carcinoma

*GREM-1* was reported to be the most consistently expressed gene at a higher level in basal cell carcinoma stromal cells compared with non-tumor skin [39]. This was further supported by Gremlin-1 expression in vivo. Meanwhile, in vitro, Gremlin-1 was reported to promote the proliferation of cultured basal cell cancer cells [39]. Hence, BMP antagonists such as Gremlin-1 may be important constituents of tumor stroma, creating a favorable TME for cancer cell survival [39].

### 6.4. Lung Cancer and Mesothelioma

In lung cancer cell line A549, the addition of Gremlin-1 induced a fibroblast-like morphology and reduced E-cadherin expression [49]. Furthermore, A549 cells incubated, or overexpressing *GREM-1,* showed an increase in the tumor volume, migration, proliferation, and invasion potential. Such effects were inhibited by the addition of the Gremlin-1 antibody [49].

Similarly, high Gremlin-1 expression was reported in mesothelioma tumor tissues and primary mesothelioma cells cultured from pleural effusion samples [62,75,76]. Further, its expression was linked to changes in the expression of integrins, MMPs, and TGF-β family signaling, which are linked to a mesenchymal invasive phenotype [62]. Studies revealed Gremlin-1 to have the ability to promote mesothelioma cell sprouting and invasion in 3D collagen and matrigel matrices [62]. The downregulation of Gremlin-1 expression inhibited cell proliferation in a mesothelioma cell line, along with downregulation of mesenchymal proteins linked to cancer EMT [75,76]. Moreover, in vivo mesothelioma xenograft experiments indicated that *GREM-1* overexpressing tumors were more vascular and had a metastatic potential [62]. Resistance to cell death induced by the chemotherapeutic agent paclitaxel was associated with high Gremlin-1 expression. In addition, it was reported that there are interactions and colocalizations of Gremlin-1 and fibrillin peptides [75]. Therefore, Gremlin-1 triggers mesothelioma invasion and metastasis and could be a potential therapeutic target, especially for overcoming drug resistance in mesothelioma.

### 6.5. Colorectal and Gastric Cancer

Despite the fact that the loss of BMP and increased expression of Gremlin-1 resulted in the development of intestinal juvenile polyps, little is known about Gremlin-1 expression and its role in colorectal cancer (CRC) [37,77]. Gremlin-1, BMP-4, and BMP-2 variations were discovered to be the cause of the formation of familial CRC. Research confirmed that abnormal epithelial Gremlin-1 expression triggered hereditary mixed polyposis syndrome (HMPS), first identified in Ashkenazi Jews [78], and colonic carcinogenesis from cells beyond the crypt base stem cell niche [37,38]. Gremlin-1 testing with a broader scope will improve the detection of hereditary polyposis and CRC, as well as the prevention and early diagnosis for mutation carriers [78].

The BMP pathway is a major barrier to the development of intestinal cancer and is commonly inhibited during the disease. However, BMP pathway components implicated in upstream pathways of BMP inhibition in CRC are still poorly understood. It was found that in the preneoplastic inflammatory milieu that precedes adenoma and carcinoma, an extracellular BMP suppression signature including Gremlin-1 was dysregulated. The BMP pathway has been demonstrated to combine with multiple colonic tumorigenesis molecular pathways in the intestinal mucosa [79]. For example, it was discovered that deactivation of the uPA/TGF-β pathway, which is a common feature of early-stage colon cancer, is linked to stronger suppression of the BMP pathway, as evidenced by BMP-4 reduction and Gremlin-1 increase, which shed more light on the contradicting roles of the TGF-β/BMP pathway in colon cancer progression [80]. Surprisingly, it has been suggested in the few studies that have been conducted so far [81,82] that the BMP pathway is completely shut down once the neoplastic lesions have transitioned to an advanced and poorly differentiated stage, particularly through blocking mutations in Smad4 and/or BMP receptors. However, the same investigations indicated that the majority of colon adenomas have an active BMP pathway [81,82]. As a result, rather than being a bimodal impact, BMP pathway inhibition appears to be an acquired skill that develops over time in the colon milieu.

BMP signalling has been thought to have tumor-suppressive properties in CRC, by lowering cancer cell growth, infiltration, and migration, as well as antagonizing EMT [83,84,85]. Gremlin-1, on the other hand, changed cancer cells’ maturation and differentiation toward a more mesenchymal-like and stem-like phenotype, possibly promoting their survival within the tumor structure [50,86]. EMT was shown to confer migratory and invasive features to tumor cells, produce cancer stem cell traits, and block apoptosis and senescence [87,88]. As a result, treatment with anti-EMT agents offer a promising tool for CRC therapeutic development.

Gremlin-1 was discovered to be expressed by intestinal smooth muscle cells in the muscularis mucosa and fibroblasts around the crypts, forming an increasing gradient toward the crypt in the normal colon. Hence, maintaining the intestinal stem cell niche in the colonic basal crypt [14]. In CRC, CAFs are thought to enhance EMT, infiltration, and spread in cancer patients, resulting in a poor prognosis. According to a recent study, stromal Gremlin-1 expression was significantly linked to increased overall and recurrence-free survival. Gremlin-1 expression was also considered a separate prognostic biomarker for both stage II and III CRCs [89]. Therefore, Gremlin-1 expression in CAFs could have an impact on the evolution of the CRC [38,90].

Recent data link Gremlin-1 expression and function to enhanced angiogenesis in the microenvironment of the colon [91]. This is accomplished by Gremlin-1 binding and phosphorylating the VEGFR2 in a BMP-independent way [51], resulting in the development of the VEGFR2/Integrin-v3 complex, which promotes angiogenic budding [92]. Moreover, Liu et al. linked the mechanism of Gremlin-1 regulating cancer formation to the BMP/SMAD and VEGF-related downstream pathway [87,93,94].

BMPs have been shown to have a role in the progression of metaplastic and dysplastic alterations in normal stomach tissue [95]. Gremlin-1 expression was confirmed in normal, metaplastic, and malignant stomach tissues, where epithelial Gremlin-1 expression was found to be linked with tumor progression and to be an independent predictor of poor survival in gastric cancer (GC) patients. Thus, Gremlin-1 could be a potential prognostic classifier in GC patients [96], similar to colon cancer [90]. Both CRC and GC cells with similar genetic and phenotypic origins are likely to have similar Gremlin-1 expression patterns [96]. In particular, Gremlin-1 was found to be selectively expressed in CRC cases with serrated morphology, which often displays gastric features [87,97].

Gremlin-1 expression was linked to a shallower tumor depth, smaller tumor size, less nodal involvement, vascular invasion, and a better five-year survival rate according to Yamasaki et al. [98]. On the contrary, another study reported that Gremlin-1 was associated with tumor development and invasion, as well as lymph node metastasis [99]. Furthermore, increased Gremlin-1 expression in GC was associated with lower overall survival and progression-free survival [99].

The molecular and cellular mechanisms underlying Gremlin-1 participation in GC disease progression is still under investigation. A study by Scherberich A et al. showed that *GREM-1* knockdown increased GC cell invasion and migration by removing the antagonistic effect on BMP-induced cancer cell invasiveness and migration [100]. Additionally, Gremlin-1 expression was reported to be associated with the expression of DNA binding proteins, including ID1, ID2, ID3, SNAI1, SNAI2, and TWIST1. This suggests an involvement of Gremlin-1 in EMT of GC but still requires further investigation in future studies [99].

### 6.6. Cholangiocarcinoma and Pancreatic Cancer

Like many other cancers, Gremlin-1 protein was found to be overexpressed in extrahepatic cholangiocarcinoma in comparison to peritumoral tissues, adenoma, and normal biliary tract [101]. Such an expression pattern was found to be correlated to poor differentiation, lymph node metastasis, invasion of surrounding tissues and organs, as well as late and advanced tumor stages. Additionally, patients with extrahepatic cholangiocarcinoma showing high Gremlin-1 expression had shorter survival time. Thus, Gremlin-1 could be a potential independent poor prognostic factor in cholangiocarcinoma patients [101].

In pancreatic cancer, there was an elevation in Gremlin-1 expression, especially in the stroma, which was correlated with survival rate and stage [102]. In addition, the activation of the pancreatic stellate cells caused an increase in Gremlin-1 expression. Co-culture experiments revealed that paracrine sonic hedgehog from the pancreatic cancer cell line AsPC-1 induced the expression of Gremlin-1 in the pancreatic stellate cells. Furthermore, silencing of *GREM-1* negatively regulated the proliferation and migration of pancreatic stellate cells as well as the proliferation, invasion, and EMT of AsPC-1 and BxPC-3 pancreatic cancer cell lines [102].

In addition, there was a close correlation between Gremlin-1 expression and favorable prognosis in pancreatic neuroendocrine tumors (NETs). Due to its pro-angiogenic properties, Gremlin-1 was found to be significantly associated with a high microvessel density [103]. Therefore, Gremlin-1 expression was correlated with tumor-associated angiogenesis and could be a novel prognostic marker and tumor suppressor in pancreatic NETs [103].

### 6.7. Glioma and Glioblastoma

In glial-tumors such as glioblastoma multiforme, *GREM-1* overexpression in the cancer stem cells compartment was found to be crucial for tumor cell growth in vitro and in vivo [104,105]. Further, in glioblastoma, Gremlin-1 was reported to bind to BMP and inhibit BMP-signaling, thereby inhibiting cellular differentiation and maintaining tumorgenicity [104,106]. Moreover, *GREM-1* overexpression in glioma cell lines induced EMT by upregulating the expression of E-Cadherin and BMP-7, and activation of TGF-β signaling [107]. Another possible signaling pathway is through Gremlin-1 binding to VEGFR2, leading to stimulation of the migration of endothelial cells and angiogenesis regulation [51,108,109]. On the other hand, *GREM-1* knockdown reduced the cell viability, promoted apoptosis, and inhibited the migration, invasion, and EMT process in the glioblastoma cell line U87-MG. Taking a closer look at the TGF signaling pathway, *GREM-1* knockdown was reported to inhibit TGF-β1-mediated activation of the Smad pathway [107].

In glioblastoma, Gremlin-1 was found to be secreted more by the CSCs component of the tumor in comparison to the other surrounding glioma cells. The maintenance of undifferentiated CSCs is an important factor in tumor growth and virulence. Furthermore, Gremlin-1 maintains CSCs by opposing the further differentiation of CSCs through the inhibition of the BMP pathways [105].

## 7. Expression and Prognostic Value of Gremlin-1 in Non-Neoplastic Diseases

Although the main focus of this review is to discuss the role and importance of Gremlin-1 in cancer initiation and progression, it is worth mentioning that the expression and potential prognostic value of Gremlin-1 have been correlated to other diseases, such as type-2 diabetes [110], nonalcoholic fatty liver disease [110], obesity [111], pulmonary arterial hypertension [112], and Parkinson’s disease. It was also associated with other processes involved in the pathophysiology of kidney diseases [113] as well as wound healing and fibrosis [32]. 

## 8. Conclusions

**Figure 3:**
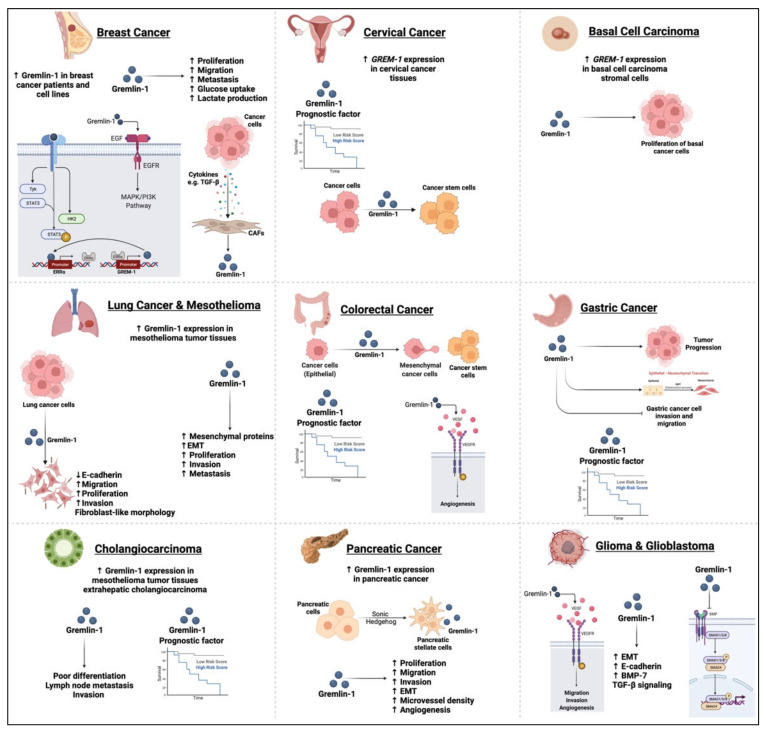
Role of Gremlin-1 in several cancer types including breast and cervical cancer, basal cell carcinoma, as well as lung, colon, gastric cancers, cholangiocarcinoma, pancreatic cancer and glioblastomas.

In this review, we shed the light on the role of one of the BMP antagonists, Gremlin-1, in the initiation and progression of various cancer types (Figure 3). Cumulatively, the studies revealed Gremlin-1 to be a key player in multiple processes associated with cancer development, including proliferation, migration, invasion, and EMT. This could be through multiple pathways, including BMP dependent and independent pathways, such as the VEGFR signaling pathway. Such findings indicate that Gremlin-1 could be a promising prognostic biomarker and therapeutic target for different malignancies. However, the exact underlying mechanisms of Gremlin-1 and its interaction with other cellular components in cancer progression need to be further explored. 

## Data Availability

Not applicable.

## Abbreviations

BMP	Bone Morphogenic Protein
BMPR	Bone Morphogenic Protein Receptor
CAFs	Cancer-Associated Fibroblasts
CRC	Colorectal Cancer
CSCs	Cancer Stem Cells
ECM	Extracellular Matrix
EGFR	Epidermal Growth Factor Receptor
EMT	Epithelial-Mesenchymal Transition
ER	Estrogen Receptor
ERRα	Estrogen-Related Receptor α
GC	Gastric Cancer
HK2	Hexokinase-2
HMPS	Hereditary Mixed Polyposis Syndrome
ID	Inhibitor of Differentiation
MAPK	Mitogen-Activated Protein Kinase
MIF	Migration Inhibitory Factor
MMPs	Matrix Metalloproteinases
NETs	Neuroendocrine Tumors
PRDC	Protein Related to DAN and Cerberus
ROS	Reactive Oxygen Species
SMAD	Small Mothers Against Decapentaplegic
SNAI1	Snail Family Transcriptional Repressor 1
STAT	Signal Transducer and Activator of Transcription
TME	Tumor Microenvironment
TGF-β	Transforming Growth Factor-beta
TNF-α	Tumor Necrosis Factor -alpha
TWIST1	Twist-related protein 1
VEGF	Vascular Endothelial Growth Factor
VEGFR	Vascular Endothelial Growth Factor Receptor
